# Exosome-enriched extracellular vesicles are associated with Getah virus RNA and transmission-related readouts *in vitro*

**DOI:** 10.3389/fcimb.2026.1838901

**Published:** 2026-06-15

**Authors:** Yinghao Liu, Decheng Yang, Weiliang Liu, Lili Wen, Lan Du, Xianfeng Zhang, Shouping Hu, Jingfei Wang, Xijun He

**Affiliations:** 1 State Key Laboratory for Animal Disease Control and Prevention, Harbin Veterinary Research Institute of Chinese Academy of Agricultural Sciences, Harbin, China; 2 Graduate School of Medicine, The University of Tokyo, Tokyo, Japan

**Keywords:** alphavirus, exosome-enriched EVs, extracellular vesicles, Getah virus, intercellular spread

## Abstract

**Background:**

Getah virus (GETV) is a mosquito-borne alphavirus of veterinary importance with potential zoonotic relevance. Extracellular vesicles (EVs) can facilitate intercellular transfer of viral components, but their involvement in GETV infection remains unclear.

**Methods:**

Exosome-enriched EV fractions were isolated from supernatants of GETV-infected HeLa, BHK21, and PK15 cells by ultracentrifugation followed by CD9 immunoaffinity enrichment (HeLa) or iodixanol density gradient fractionation (BHK21/PK15). EV-associated viral RNA and infection-related readouts were analyzed using RT-PCR, RT-qPCR, immunoblotting, immunofluorescence, transmission electron microscopy, and uptake assays.

**Results:**

Exosome-enriched EV fractions were enriched for canonical EV markers and were associated with genome-spanning GETV RNA. Exposure of recipient cells to these fractions resulted in EV uptake, increased intracellular GETV RNA levels, and viral protein expression. RNase protection assays showed that the RNA signal was resistant to RNase treatment unless membranes were disrupted, consistent with membrane-protected RNA. In donor HeLa cells, GW4869 treatment reduced EV recovery and EV-associated GETV RNA levels and decreased transmission-related readouts in recipient cells, while having limited effects on cell-free virus replication in donor cells.

**Discussion:**

Together, these findings support the existence of an EV-associated route that may contribute to GETV transmission-related processes *in vitro*. However, complete physical separation of EVs from virions and the mechanism of genome delivery require further validation.

## Introduction

Getah virus (GETV), a mosquito-borne arbovirus of the Alphavirus genus (Togaviridae family), was first isolated from Culex gelidus mosquitoes in Malaysia in 1955 (Karabatsos, 1978; Barboza et al., 2008). Over the past seven decades, GETV has expanded geographically, infecting primary hosts such as pigs, cattle, horses, and foxes, with emerging reports suggesting broader host ranges, including monkeys, kangaroos, goats, rabbits, and wild birds (Wada et al., 1982; Lu et al., 2019; Shi et al., 2019; Li et al., 2022; Huang et al., 2025; Shen et al., 2025). GETV infections cause arthritis, fever, and reproductive disorders, leading to significant economic losses in animal husbandry, particularly in pigs (Wen et al., 2007; Xing et al., 2020). In horses, symptoms include anorexia, lymphadenopathy, hind limb edema, and urticarial rash (Lu et al., 2019). More concerningly, GETV-neutralizing antibodies have been detected in human serum in regions including China and Australia (Kanamitsu et al., 1979; Li et al., 1992). Moreover, the antibody titers in febrile individuals are significantly higher than those in healthy populations (Li et al., 2022), suggesting human exposure and a potential risk of human infection. GETV therefore warrants further investigation.

Like other alphaviruses, GETV is an enveloped, single-stranded positive-sense RNA virus with an 11–12 kb genome comprising two open reading frames (ORFs), a 5’ methylated cap, a 26S subgenomic promoter, and a 3’ poly-A tail (Button et al., 2020). The 5’ ORF encodes four non-structural proteins (nsP1-4) forming the viral replicase complex responsible for genome transcription, replication, polyprotein cleavage, and RNA capping, while the 3’ ORF encodes structural proteins (C, E3, E2, 6K, E1) (Li et al., 2010; Button et al., 2020; Wang et al., 2022). Mature virions (~70 nm) consist of an icosahedral nucleocapsid (240 capsid proteins) and an outer envelope with 80 E1/E2 trimeric spikes, both exhibiting T = 4 symmetry (Wang et al., 2022) (see **Supplementary Figure S1**).

Small extracellular vesicles (sEVs), including exosomes, are membrane-bound vesicles typically 30–150 nm in diameter that are released through endosomal pathways. They are formed during maturation of early endosomes into late endosomes and multivesicular bodies (MVBs), and are released upon MVB-plasma membrane fusion via ESCRT-dependent or ESCRT-independent mechanisms (Ghossoub et al., 2014; Juan and Fürthauer, 2018). sEVs carry diverse biomolecules (proteins, RNAs, and lipids) and mediate intercellular communication and physiological homeostasis (Raposo and Stoorvogel, 2013; Zhang et al., 2015; Kalluri and LeBleu, 2020). Because operational isolation methods enrich for exosome markers but may include other small EV subtypes, we refer to our preparations as “exosome-enriched EV fractions” throughout. Given the overlap between viral life cycles and EV biogenesis, many viruses exploit EV pathways to facilitate spread or modulate host responses. For example, exosomes from hepatitis C virus (HCV)-infected cells can transfer immunomodulatory viral RNA to neighboring immune cells, triggering the expansion of myeloid-derived suppressor cells (MDSCs) and promoting TFR differentiation while inhibiting TFH function (Wang et al., 2018). Enterovirus 71 (EV71)-infected cell-derived exosomes carry miR-146a, which facilitates viral RNA replication in recipient cells by inhibiting type 1 interferon signaling (Fu et al., 2017). Exosomes isolated from porcine reproductive and respiratory syndrome virus (PRRSV)-infected cells contain the viral genome and have been reported to transmit infection-related signals to recipient cells (Wang et al., 2018). Recently, Chikungunya virus (CHIKV), another member of the genus Alphavirus, has been reported to utilize EVs for transmission (Le et al., 2022). Despite extensive research highlighting roles for EVs in viral transmission, the potential involvement of exosome-enriched EV fractions in GETV infection remains unexplored.

In this study, we isolated and characterized exosome-enriched EV fractions derived from GETV-infected cells and assessed their association with viral RNA and infection-related readouts in recipient cells. We observed full-length GETV genomic RNA in exosome-enriched EV fractions and found that exposure of recipient cells to these fractions was associated with viral RNA accumulation and viral protein expression. Similar EV-associated transmission-related signals were observed in PK15 and BHK21 cells, suggesting that EV association is not restricted to a single mammalian cell line. Together, our findings provide evidence consistent with an EV-associated route that may contribute to GETV dissemination *in vitro* and motivate future studies to define the physical relationship between EVs and virions and the mechanism of entry and genome delivery.

## Materials and methods

### Cell culture and virus

HeLa (human cervical carcinoma, ATCC CCL-2), BHK21 (baby hamster kidney, ATCC CCL-10), and PK15 (porcine kidney, ATCC CCL-33) cells were maintained in DMEM (Gibco, USA) supplemented with 10% exosome-depleted fetal bovine serum (System Biosciences, USA) and 1% penicillin–streptomycin at 37 °C in 5% CO_2_. The GETV strain SC483 used in this study was isolated from clinically healthy pigs in Sichuan Province in 2018 by our laboratory, GETV strain SC483 (GenBank: MN478486). For infection and EV collection, cells were cultured in DMEM containing 2% exosome-depleted FBS. GETV strain SC483 (GenBank: MN478486) was propagated in BHK21 cells, and viral titers were determined by TCID_50_. Unless otherwise specified, infections were performed at MOI = 1.0; after adsorption for 1 h, inocula were removed, cells were washed with PBS, and fresh medium containing 2% exosome-depleted FBS was added. Experiments were conducted under Biosafety Level 2 conditions (protocol #2023-GETV-01).

### Exosome isolation and purification

To prepare exosome-enriched EV fractions, culture supernatants from GETV-infected or mock-infected cells were collected and sequentially centrifuged at 300 × g for 5 min, 3000 × g for 10 min, and 10,000 × g for 30 min to remove cells and debris, followed by filtration through 0.2-µm membranes. Clarified supernatants were ultracentrifuged at 120,000 × g for 70 min at 4 °C using an SW28 Ti rotor (Beckman Coulter) to pellet crude EVs. Pellets were washed once in PBS and resuspended in PBS. For HeLa-derived preparations, crude pellets were further enriched for exosome markers by CD9 immunoaffinity capture using Protein G Dynabeads (Invitrogen) coupled to anti-CD9 antibody (Santa Cruz, sc-13118). Briefly, 50 µL Dynabeads were washed with 500 µL PBS to remove storage buffer and then incubated with 20 µg anti-CD9 antibody diluted in 500 µL PBS for 30 min at room temperature with rotation. Beads were washed twice with PBS and subsequently incubated overnight at 4 °C with crude EV preparations resuspended in 500 µL PBS under rotation. After incubation, beads were washed 3 times with PBS, and bound EVs were eluted with 50 µL pre-chilled 100 mM glycine-HCl (pH 3.0). Eluates were immediately neutralized with 1 M Tris-HCl (pH 8.5) before downstream analyzes. For BHK21 and PK15 preparations, crude EV pellets were further resolved by iodixanol density gradients as described below.

### OptiPrep density gradient exosome isolation

Due to species-specific differences in antibody compatibility, distinct EV enrichment methods were employed for human-derived versus non-human cell lines. Crude EV pellets from BHK21 or PK15 supernatants were overlaid onto discontinuous 10–40% iodixanol (OptiPrep, Sigma-Aldrich) gradients prepared in PBS (3 mL each of 10%, 20%, 30%, and 40% iodixanol) and ultracentrifuged at 120,000 × g for 16 h at 4 °C using an SW41 Ti rotor. Twelve 1-mL fractions were collected sequentially from the top of the gradient. For immunoblot analysis, fractions were diluted 5-fold in PBS and pelleted by ultracentrifugation at 120,000 × g for 2 h at 4 °C. For functional assays, fractions enriched for EV markers (fractions 4–7) were pooled for downstream functional assays, washed once in PBS by ultracentrifugation, and resuspended in PBS. Pellets intended for protein analysis were lysed in RIPA buffer.

### Western blot analysis

Proteins (10 µg) were resolved on 12% SDS-PAGE and transferred to PVDF membranes. Membranes were blocked (5% non-fat milk, 1 h) and incubated with primary antibodies (4 °C, overnight): anti-CD63 (Abcam, ab231975, 1:1000), anti-CD9 (Santa Cruz, sc-13118, 1:500), anti-TSG101 (Santa Cruz, sc-7964, 1:500), anti-HSP70 (Abcam, ab5442, 1:2000), anti-Alix (Cell Signaling, 2171s, 1:1000), anti-GRP94 (Cell Signaling, 2104s, 1:1000), anti-GETV CP (in-house, 1:500). HRP-conjugated secondary antibodies (Thermo Scientific, anti-rabbit 31460, anti-mouse 31430, 1:5000) were applied (room temperature, 1 h). Bands were visualized with ECL reagent (Biosharp, BL520A).

### Transmission electron microscopy

Exosomes or virions (20 µL) were fixed in 2.5% glutaraldehyde, applied to glow-discharged carbon-coated grids, negatively stained with 2% phosphotungstic acid (30 s), and imaged on a Hitachi H-7650 TEM at 80 kV.

### Nanoparticle tracking analysis

Exosomes were diluted 1:100 in PBS and analyzed using ZetaVIEW S/N 252 (3 measurements, 11 positions).

### EV labeling and uptake assay

Exosome-enriched EV fractions were fluorescently labeled using the PKH26 Red Fluorescent Cell Linker Kit (Sigma-Aldrich) according to the manufacturer’s instructions with minor modifications. Briefly, EV suspensions were mixed with PKH26 dye in Diluent C for 5 min at room temperature, and the reaction was quenched with exosome-depleted serum. Labeled EVs were washed to remove unincorporated dye by ultracentrifugation at 120,000 × g for 70 min at 4 °C and resuspended in PBS. Recipient HeLa cells were incubated with PKH26-labeled EVs for 8 h, washed 3 times with PBS, fixed with 4% paraformaldehyde, and stained with phalloidin and DAPI. Images were acquired by confocal microscopy.

### EV exposure assays and nuclease protection

Unless otherwise indicated, recipient cells were exposed to exosome-enriched EV fractions resuspended in PBS, or to cell-free GETV (MOI = 1.0) as a positive infection control. EV inputs were standardized across conditions by applying equal volumes of EV suspensions generated from matched volumes of donor-cell conditioned medium that were processed in parallel. For EV-associated transmission experiments assessing GW4869, the inhibitor (10 µM) or DMSO vehicle control was applied to donor (virus-infected) cells during the post-adsorption period before supernatant harvest for EV isolation; recipient cells were not treated with GW4869. For nuclease protection assays, EV preparations were treated with RNase A (10 µg/mL, 30 min, 37 °C) in the absence or presence of Triton X-100 (0.1%, 10 min) prior to incubation with recipient cells. Cells and/or supernatants were collected at the indicated time points for downstream readouts, including RT-qPCR, immunoblotting, and immunofluorescence.

### Virus titration (TCID_50_)

Infectious virus titers were determined by the 50% tissue culture infectious dose (TCID_50_) assay on BHK21 cells. Briefly, BHK21 cells were seeded in 96-well plates and cultured to near confluence. Virus-containing supernatants were serially 10-fold diluted in DMEM, and 100 µL of each dilution was added to BHK21 monolayers in 8 replicate wells per dilution. Cells were incubated at 37 °C for 72 h, after which cytopathic effect (CPE) was scored microscopically, and TCID_50_ values were calculated using the Reed–Muench method.

### RNA extraction, reverse transcription, and RT-qPCR

RNA was extracted using the Simply P Total RNA Extraction Kit (Bioer, BSC52M1). Reverse transcription was used with the PrimeScript RT reagent Kit (Takara, RR047A). RT-qPCR was performed using TB Green^®^ Premix Ex Taq^™^ (Tli RNaseH Plus) (Takara, RR820A) on a QuantStudio 3 Real-Time PCR System (Applied Biosystems, USA) under standard cycling conditions. GETV nsp1 primers were 5’-AGGTGTCAGGACGGCGTATT-3’ and 5’-ATCTGCCCAGTTGGTCGAGT-3’. Absolute quantification of GETV RNA was performed using serial dilutions of a synthetic RNA plasmid (pGETV-nsp1, in-house) as a standard (GenBank: MN478486, nsp1 region). Ten overlapping fragments covered the GETV genome for RT-PCR (see **Supplementary Table S1** for primers).

### Indirect immunofluorescence assay

Cells were fixed with 4% paraformaldehyde for 30 min at room temperature, washed 3 times with PBS, and permeabilized with 0.1% Triton X-100 for 30 min at room temperature. After washing with PBS, cells were blocked with 5% skimmed milk in PBS for 1 h at room temperature, then incubated with anti-GETV E1 monoclonal antibody (in-house, 1:500 dilution in blocking buffer) for 1 h at room temperature. Following 3 times PBS washes, Alexa Fluor-conjugated anti-rabbit antibodies (Invitrogen, 1:1000) were applied for 1 h at room temperature in the dark. Cells were washed 3 times with PBS and counterstained with DAPI. Images were captured with an EVOS M5000 Imaging System (Thermo Fisher Scientific).

### Cell viability assay

Cell viability was measured with Cell Counting Kit-8 (CCK-8; Dojindo, CK04) according to the manufacturer’s instructions. Briefly, cells were seeded in 96-well plates at 1 × 10^3cells per well and allowed to adhere for 12 h. Cells were then treated with GW4869 (Sigma-Aldrich, D1692) at indicated concentrations (0–20 µM) for 36 h. Subsequently, 10 µL CCK-8 reagent was added to each well containing 100 µL culture medium, and cells were incubated for 1 h at 37 °C. Absorbance at 450 nm was measured with a microplate reader (Thermo Fisher Scientific).

### Statistical analysis

Data normality was verified (Shapiro-Wilk test). One-way ANOVA with Bonferroni’s multiple comparison tests was performed using GraphPad Prism. P < 0.05 was significant (****p < 0.0001, ***p < 0.001, **p < 0.01, *p < 0.05).

## Results

### Isolation and characterization of exosome-enriched EV fractions from GETV-infected cells

**Figure 1:**
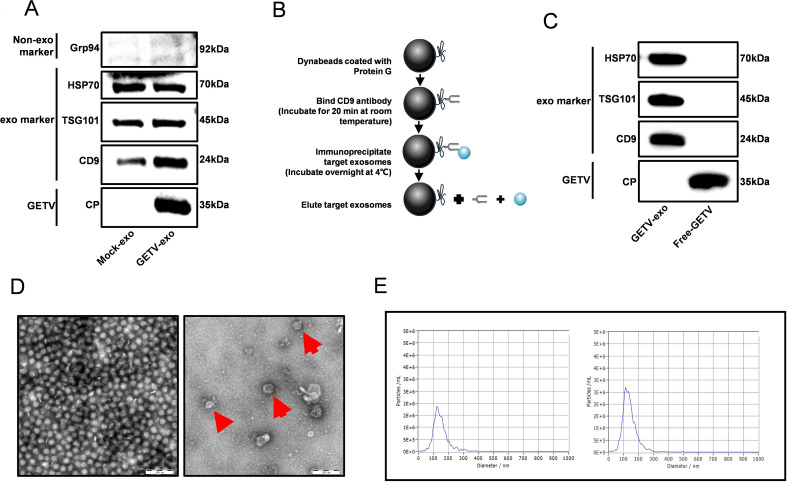
Isolation and characterization of exosome-enriched EV fractions from GETV-infected cells. **(A)** Immunoblot of crude EV pellets from mock- or GETV-infected HeLa cells for EV markers (HSP70, CD9, TSG101), endoplasmic reticulum marker (GRP94), and GETV capsid protein (CP). **(B)** Schematic of CD9 immunomagnetic bead enrichment for HeLa-derived EV preparations. **(C)** Immunoblot of CD9-enriched EV fractions and cell-free GETV virions for EV markers and GETV CP. **(D)** Negative-stain TEM of cell-free GETV (left) and CD9-enriched EV fractions (right). **(E)** NTA particle size distributions of EV preparations from mock (left) and GETV-infected (right) HeLa cells.

Exosome-enriched EV fractions were isolated from HeLa cells infected with GETV (MOI = 1.0) in DMEM containing 2% exosome-depleted FBS (System Biosciences, USA). At 36 hpi, culture supernatants were processed by differential centrifugation and ultracentrifugation to yield crude sEV pellets, which were evaluated by immunoblotting. Crude EV preparations from GETV-infected cells contained canonical EV markers (CD9, HSP70, TSG101) but also detectable GETV capsid protein (CP), consistent with co-isolation of virus-containing material (**Figure 1A**). Negative-stain TEM of crude preparations showed heterogeneous particulate material, underscoring the challenge of separating EVs from alphavirus particles of similar size and density (**Supplementary Figure S2**). To enrich for exosome markers and reduce co-isolated virus, HeLa sEVs were further purified using CD9 immunomagnetic capture (**Figure 1B**). After CD9 enrichment, CP was below the detection limit by immunoblot in the EV fraction (**Figure 1C**). Approximately 3% of detectable extracellular GETV RNA was associated with the EV fraction (**Supplementary Figure 4**). TEM provided qualitative vesicle-like morphology (approximately 100 nm) for the enriched EV fraction and a distinct morphology for free virions (approximately 70 nm) (**Figure 1D**). Nanoparticle tracking analysis (NTA) showed particle size distributions in the expected range for small EVs (approximately 30–200 nm, with a mode around 110 nm) (**Figure 1E**). Together, these results indicate that our workflow yields exosome-enriched EV fractions with CP below the detection limit by immunoblot, while acknowledging that complete physical separation of EVs and virions is intrinsically challenging.

### Exosome-enriched EV fractions from GETV-infected cells are associated with viral RNA

**Figure 2:**
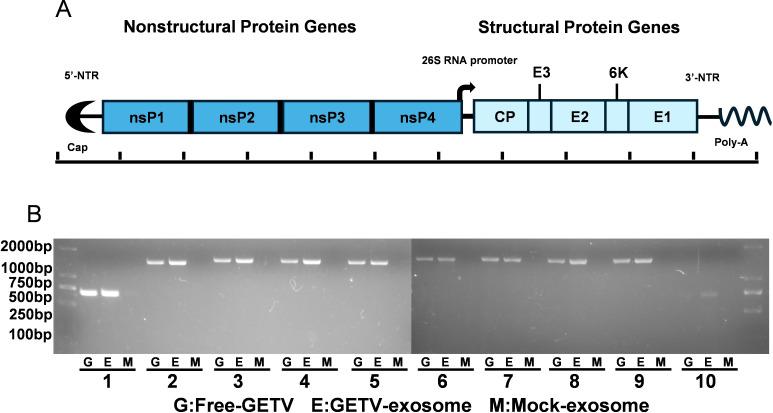
Genome-spanning GETV RNA detected in exosome-enriched EV fractions. **(A)** Schematic of the GETV genome (SC483 strain) segmented into 10 overlapping RT-PCR fragments. **(B)** RT-PCR detection of GETV RNA fragments in exosome-enriched EV fractions from GETV-infected or mock-infected HeLa cells; cell-free GETV served as a positive control.

To determine whether exosome-enriched EV fractions from GETV-infected HeLa cells were associated with viral RNA, total RNA was extracted and analyzed by RT-PCR. Given the length and secondary structure of the GETV genome (approximately 11.7 kb), amplification as a single amplicon is technically challenging. Therefore, the genome was divided into 10 overlapping fragments based on the SC483 strain sequence (**Figure 2A**). All 10 fragments were detected in exosome-enriched EV fractions from GETV-infected cells but not in mock-infected controls (**Figure 2B**), consistent with the presence of genome-spanning GETV RNA in these EV preparations. Free GETV virions served as positive controls.

### Exosome-enriched EV fractions are associated with transmission-related infection readouts in recipient cells

**Figure 3:**
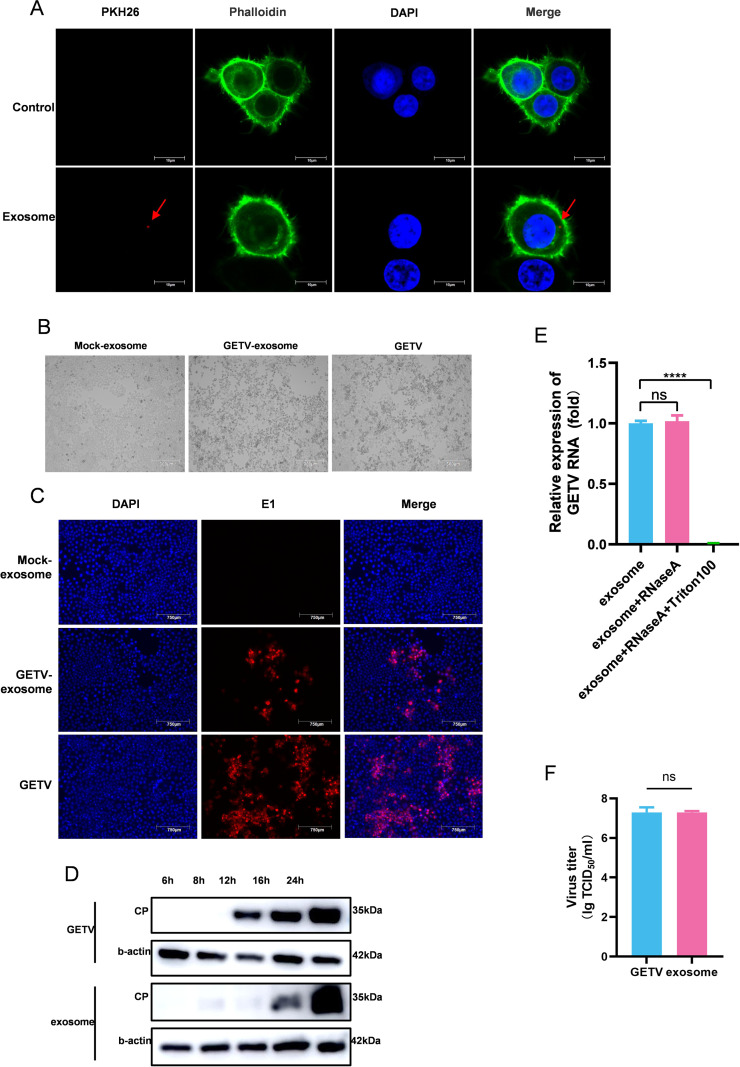
Exosome-enriched EV fractions are associated with infection-related readouts in recipient HeLa cells. **(A)** Confocal microscopy showing uptake of PKH26-labeled EV fractions (red) by recipient HeLa cells; F-actin was stained with phalloidin (green) and nuclei with DAPI (blue). **(B)** Bright-field microscopy at 24 h after exposure to EV fractions or infection with cell-free GETV (MOI = 1.0). **(C)** Indirect immunofluorescence assay (IFA) detecting GETV E1 protein at 24 h after exposure or infection. **(D)** Immunoblot detection of GETV CP in recipient HeLa cells at the indicated times after exposure to EV fractions or infection with cell-free virus. **(E)** RT-qPCR detection of GETV nsp1 in recipient HeLa cells after exposure to EV fractions pretreated with RNase A (± Triton X-100). **(F)** TCID_50_ analysis of supernatants collected from cells infected with free GETV or EV-associated GETV. ****p < 0.0001; ns, not significant.

To assess EV-associated transmission-associated readouts, we tested uptake and downstream infection markers in naive HeLa cells. PKH26-labeled exosome-enriched EV fractions were taken up by HeLa cells, as shown by confocal microscopy with phalloidin-labeled actin and DAPI-stained nuclei (**Figure 3A**), and additional wider-field representative uptake images are provided in **Supplementary Figure 5**. At 24 h after exposure, EV-treated cells displayed cytopathic changes (granular degeneration and swelling) similar to those observed after infection with cell-free GETV (MOI = 1.0) (**Figure 3B**). Immunofluorescence detected GETV E1 protein in EV-treated cells (**Figure 3C**), and immunoblotting detected CP expression at 6–24 h after exposure (**Figure 3D**). To assess whether the detected RNA signal was membrane-protected, EV fractions were treated with RNase A (10 µg/mL, 30 min, 37 °C) alone or in combination with Triton X-100 (0.1%, 10 min) prior to addition to recipient cells. RNase A treatment alone did not substantially reduce the subsequent nsp1 RNA signal in recipient cells, whereas RNase A plus Triton X-100 markedly reduced the signal (**Figure 3E**), consistent with membrane-protected RNA in the EV preparation. Because EVs and virions can share similar sensitivities to detergent and nuclease treatment, these results should be interpreted as supportive evidence rather than definitive proof of luminal RNA packaging. To further determine whether EV-associated GETV could establish productive infection, culture supernatants collected from EV-treated cells were subjected to TCID_50_ analysis. Comparable viral titers were observed between the EV-associated GETV group and the cell-free GETV group, with no statistically significant difference detected (**Figure 3F**). These findings demonstrate that EV-associated GETV retains infectivity and is capable of mediating productive infection in recipient cells.

### Inhibition of EV release in donor cells reduces EV-associated transmission-related readouts in HeLa

**Figure 4:**
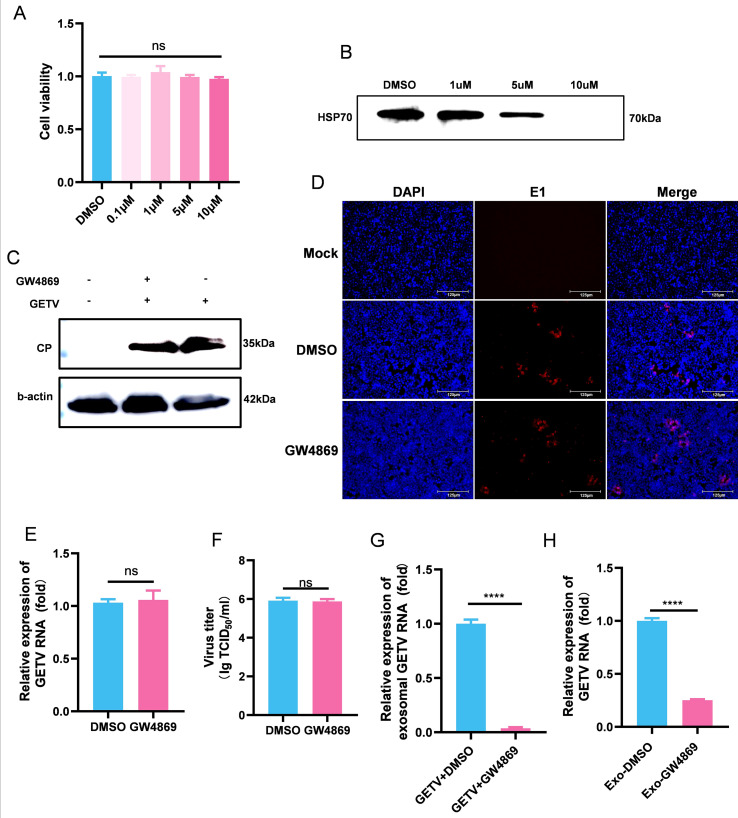
Inhibition of EV release in donor HeLa cells is associated with reduced EV-associated transmission-related readouts. **(A)** CCK-8 assay of HeLa cell viability after GW4869 treatment. **(B)** Immunoblot detection of HSP70 in EV fractions collected from GW4869-treated donor HeLa cells. **(C)** Immunoblot detection of GETV CP in donor HeLa cells with or without GW4869. **(D)** IFA detecting E1 in donor HeLa cells treated with GW4869 or DMSO. **(E)** TCID_50_ and RT-qPCR quantification of cell-free virus titers and intracellular GETV RNA in donor HeLa cells. **(F)** RT-qPCR quantification of GETV RNA in EV fractions collected from GW4869-treated versus DMSO-treated donor cells. **(G)** RT-qPCR quantification of GETV RNA in recipient HeLa cells after exposure to EV fractions collected from GW4869-treated versus DMSO-treated donors. ****p < 0.0001; ns, not significant.

To further investigate the contribution of EV release to EV-associated infection-associated readouts, GETV-infected donor HeLa cells were treated with GW4869, a neutral sphingomyelinase inhibitor commonly used to reduce EV release. A CCK-8 assay confirmed a lack of overt cytotoxicity at the indicated concentrations (**Figure 4A**). Immunoblotting showed a dose-dependent reduction of HSP70 in EV fractions collected from GW4869-treated donor cells (**Figure 4B**). A similar reduction was observed for Alix (**Supplementary Figure 6**), consistent with reduced EV recovery under these conditions. When donor HeLa cells were infected with GETV (MOI = 1.0) and treated with 10 µM GW4869, cytopathic effects were comparable to DMSO-treated controls (**Supplementary Figure S3**). Consistently, CP and E1 protein expression in donor cells was similar between GW4869 and DMSO groups (**Figures 4C, D**), and GW4869 did not measurably change genome replication (RT-qPCR) or viral titers (TCID_50_) in donor cells compared with DMSO controls (**Figure 4E**). In contrast, GETV RNA levels measured in EV fractions collected from GW4869-treated donor cells were reduced (**Figure 4F**), and naive HeLa cells exposed to these EV fractions showed lower GETV RNA levels than cells exposed to EV fractions from DMSO-treated donors (**Figure 4G**). Together, these observations are consistent with a role for donor-cell EV release in EV-associated transmission-associated readouts in HeLa cells, while cell-free virus replication in donor cells is relatively insensitive to GW4869 under these conditions.

### EV-associated infection-related readouts are observed across multiple mammalian cell types

**Figure 5:**
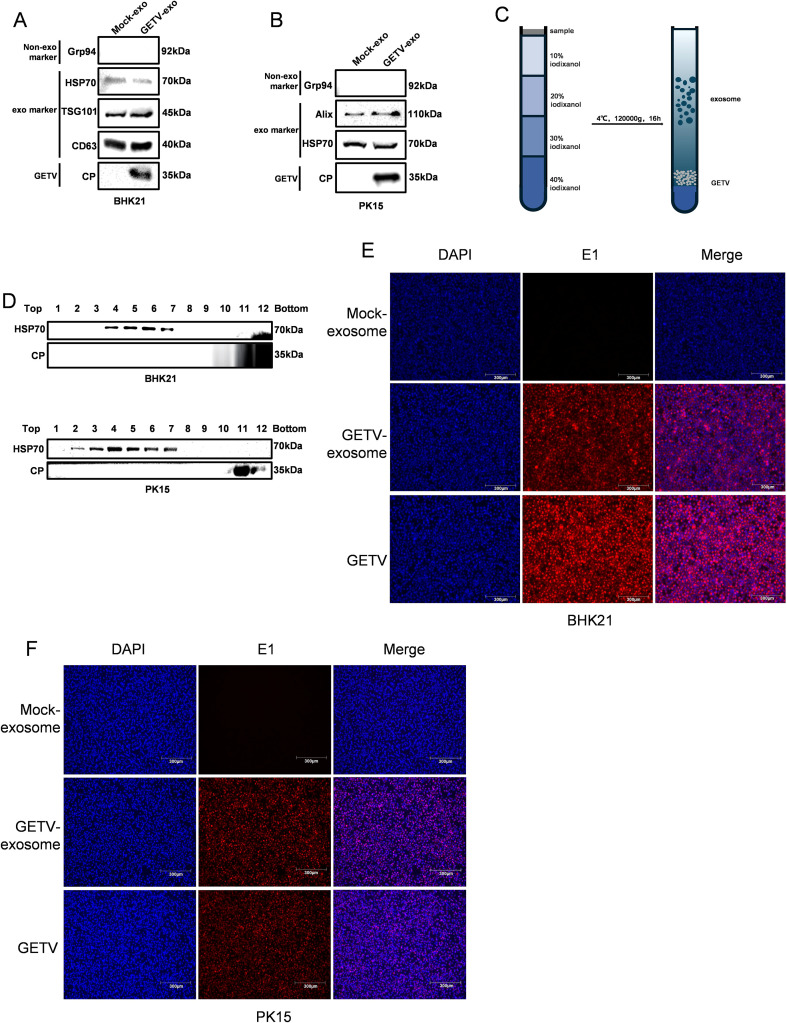
EV-associated transmission-related readouts in BHK21 and PK15 cells. **(A)** Immunoblot detection of EV markers in crude EV pellets from mock-infected or GETV-infected BHK21 cells (HSP70, CD63, TSG101, GRP94) and GETV CP. **(B)** Immunoblot detection of EV markers and GETV CP in crude EV pellets from mock-infected or GETV-infected PK15 cells. **(C)** Schematic of OptiPrep (iodixanol) density gradient fractionation. **(D)** Immunoblot detection of HSP70 and GETV CP across 12 gradient fractions from GETV-infected cells (fractions 4–7: EV marker-enriched; fractions 11–12: CP-enriched). **(E)** IFA detecting E1 in BHK21 cells exposed to pooled EV-enriched fractions (fractions 4–7) or infected with cell-free GETV (MOI = 1.0). **(F)** IFA detecting E1 in PK15 cells exposed to pooled EV-enriched fractions (fractions 4–7) or infected with cell-free GETV (MOI = 1.0).

Having observed EV-associated downstream viral readouts in HeLa cells, we investigated whether similar findings could be detected in additional mammalian cell types. We therefore examined BHK21 (baby hamster kidney) and PK15 (porcine kidney) cells. Exosome-enriched EV fractions recovered from GETV-infected (MOI = 1.0) BHK21 and PK15 cells contained EV markers (HSP70, CD63, TSG101) and lacked the endoplasmic reticulum marker GRP94 (**Figures 5A, B**). To better separate EV-associated material from virions, preparations were further resolved by OptiPrep iodixanol density gradient centrifugation (10-40%) (**Figure 5C**). Immunoblotting of gradient fractions showed HSP70 enrichment in fractions 4-7, whereas CP was enriched in fractions 11-12 (**Figure 5D**). Fractions 4–7 were pooled as exosome-enriched EV fractions and incubated with naive BHK21 or PK15 cells. Immunofluorescence detected GETV E1 protein in EV-exposed cells, similar to cells infected with cell-free GETV (MOI = 1.0) (**Figure 5E** for BHK21; **Figure 5F** for PK15). GW4869 treatment increased infection readouts in BHK21 and PK15 cells (**Supplementary Figure 2**), indicating that the net effect of EV modulation can be cell-type dependent. Collectively, these data suggest that EV association and EV-exposure readouts are not restricted to a single mammalian cell line; however, additional work in insect vector cells will be required to establish relevance for arboviral transmission.

### GW4869 treatment is associated with increased GETV replication readouts in BHK21 and PK15 cells

**Figure 6:**
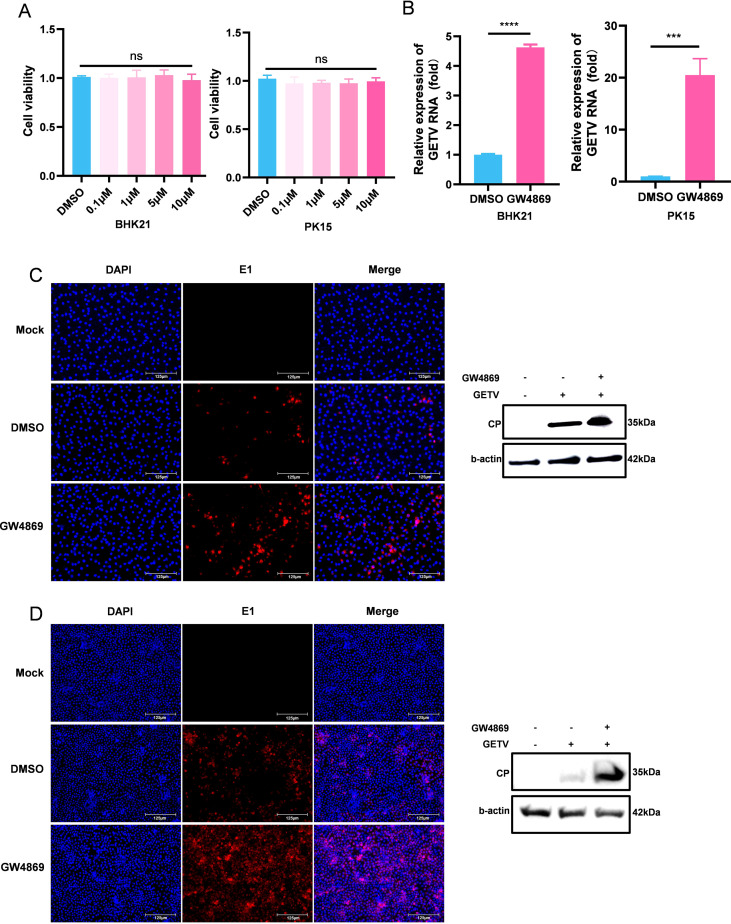
GW4869 treatment is associated with increased GETV replication readouts in BHK21 and PK15 cells. **(A)** CCK-8 assay of BHK21 and PK15 cell viability after GW4869 treatment. **(B)** TCID_50_ and RT-qPCR quantification of cell-free virus titers and intracellular GETV RNA in GW4869- or DMSO-treated, GETV-infected BHK21 and PK15 cells. **(C)** BHK21: IFA detecting E1 and immunoblot detection of CP in GETV-infected cells treated with GW4869 or DMSO. **(D)** PK15: IFA detecting E1 and immunoblot detection of CP in GETV-infected cells treated with GW4869 or DMSO. ***p < 0.001; ****p < 0.0001; ns, not significant.

To examine whether pharmacological inhibition of EV release alters GETV infection readouts in BHK21 and PK15 cells, GW4869 was applied during cell-free infection. A CCK-8 assay confirmed no obvious cytotoxicity at 0–10 µM GW4869 (**Figure 6A**). Under these conditions, GETV-infected cultures (MOI = 1.0) treated with 10 µM GW4869 displayed more pronounced cytopathic effects than DMSO-treated controls (**Supplementary Figure S3**). Consistently, viral titers and genome replication measured by TCID_50_ and RT-qPCR were increased in GW4869-treated cultures relative to controls (**Figure 6B**). Immunoblotting and immunofluorescence further showed higher levels of viral protein expression in the GW4869-treated group (**Figures 6C, D**). Together, these data indicate that GW4869 treatment is associated with increased GETV replication readouts in BHK21 and PK15 cells under the conditions tested.

## Discussion

**Figure 7:**
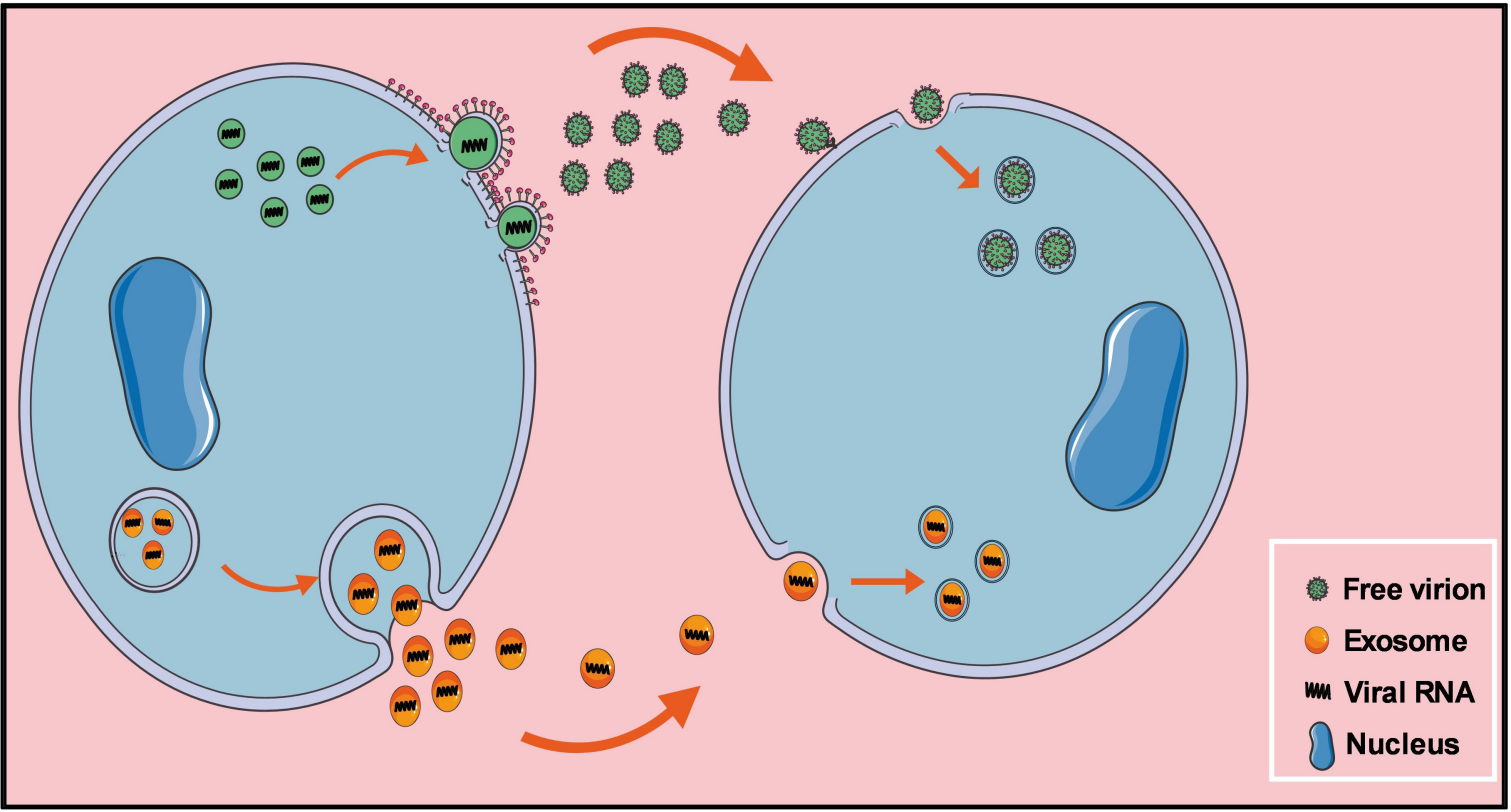
Proposed working model for EV-associated GETV transmission-related processes *in vitro* and points requiring further validation.

Exosome-enriched EV fractions are important mediators of intercellular communication and can transfer proteins, lipids, and RNAs between cells (Wolfers et al., 2001; Colombo et al., 2014; Sung et al., 2019). The ability of EVs to carry diverse biomolecules provides an opportunity for viruses to exploit this pathway to enhance spread or modulate host responses. Indeed, multiple viruses, including HBV, HCV, and herpes simplex virus type 1 (HSV-1), have been reported to use EV-associated routes to transfer viral components or influence immunity (Meckes et al., 2010; Bukong et al., 2014; Kalamvoki et al., 2014; Felli et al., 2017; Yang et al., 2017; Zhang et al., 2019; Bao et al., 2024). In the present study, we provide evidence that exosome-enriched EV fractions recovered from GETV-infected cells are associated with genome-spanning GETV RNA and that exposure of recipient cells to these fractions is associated with viral RNA accumulation and viral protein expression *in vitro*. These observations support the possibility that EV association may contribute to GETV transmission-related processes *in vitro* and motivate further mechanistic studies (**Figure 7**).

Despite significant progress in understanding virus-EV interactions, efficiently separating EVs from viruses remains challenging because EVs and virions can overlap in size and buoyant density (Gorgzadeh et al., 2024). Common EV isolation techniques, including ultracentrifugation, ultrafiltration, size-exclusion chromatography, precipitation-based methods, and immunoaffinity capture, each have limitations when applied to virus-containing samples (Rezaie et al., 2021; Lai et al., 2022). In our study, ultracentrifugation alone was insufficient to separate EV-associated material from GETV-derived material (**Figure 1A**), likely reflecting overlapping biophysical properties. To enrich for exosome markers and reduce co-isolated virus, we used a two-step approach consisting of ultracentrifugation followed by CD9-based immunomagnetic capture (HeLa, **Figure 1B**) or iodixanol density gradient centrifugation (BHK21 and PK15, **Figure 5C**). This workflow yielded EV fractions enriched for canonical EV markers and with CP below the detection limit by immunoblot, while TEM and NTA provided qualitative morphology and particle size distributions consistent with small EVs (**Figures 1D, E**). Because additional purification steps can reduce EV yield and potentially alter function (Muller et al., 2014; Paolini et al., 2016), we assessed uptake by recipient cells and observed efficient internalization of PKH26-labeled EV fractions (**Figure 3A**), consistent with retention of uptake competency.

Interestingly, we observed cell-type-specific outcomes upon pharmacological inhibition of EV release. In HeLa cells, reducing EV recovery from donor cells with GW4869 was associated with decreased EV-associated downstream viral readouts in recipient cells (**Figure 4G**), whereas cell-free virus replication in donor cells was not measurably altered (**Figure 4E**). In contrast, GW4869 treatment of GETV-infected BHK21 and PK15 donor cells increased viral replication readouts (**Figure 6B**), suggesting that EV modulation can have distinct net effects depending on cellular context. One possibility is that EV-associated factors may differentially modulate infection depending on cellular context, although the precise mechanisms remain unresolved. We note that GW4869 can exert pleiotropic effects beyond EV biogenesis, and orthogonal genetic perturbations of EV pathways would be valuable for future validation.

Limitations of this study should be noted. First, although our EV fractions were enriched for canonical EV markers and GETV CP was not readily detectable by immunoblot after enrichment, complete physical separation of EVs from virions cannot be guaranteed for alphaviruses because of overlapping size and buoyant density. Thus, the observed findings should be interpreted as evidence supporting EV-associated transmission of GETV rather than definitive proof that intact virions within EVs. Second, the enrichment strategies used in this study yield heterogeneous EV-enriched preparations rather than purified exosome populations; in particular, CD9-based immunoenrichment captures only a CD9-positive EV subset and does not encompass the full heterogeneity of extracellular vesicles. Negative-stain TEM provides qualitative morphology but does not resolve internal cargo. Although capsid protein was not detected in purified EV fractions, the presence of other viral structural components, such as envelope proteins (e.g., E1), was not specifically assessed. Therefore, higher-resolution imaging (e.g., cryo-EM, immunogold labeling) and orthogonal purification strategies would be needed in future studies to localize viral components. Third, EV inputs were standardized by processed donor supernatant volume; future work should additionally normalize by particle number and/or EV protein content. Finally, the entry and genome-delivery mechanism remains unresolved, and pharmacologic perturbation using GW4869 should be interpreted cautiously given its potential EV-independent effects on cellular lipid metabolism and viral replication pathways. Experiments using receptor-deficient or receptor-blocked cells, single-molecule RNA imaging, and genetic perturbation of EV biogenesis would strengthen mechanistic inference.

## Data Availability

The datasets presented in this study can be found in online repositories. The names of the repository/repositories and accession number(s) can be found below: Zenodo (https://doi.org/10.5281/zenodo.16731878) (Liu et al., 2025).

## Supplementary material

Supplementary Figure 1 GETV virion structure.

Supplementary Figure 2 TEM of negatively stained crude EV pellets.

Supplementary Figure 3 GW4869 effects in different cell lines.

Supplementary Figure 4 Quantification of the proportion of total extracellular GETV RNA with exosome-enriched EV preparations.

Supplementary Figure 5 Wider-field representative images of PKH26-labeled exosome-enriched EV uptake by recipient HeLa cells.

Supplementary Figure 6 GW4869 treatment is associated with reduced recovery of Alix in exosome-enriched EV preparations.

Supplementary Table 1 RT-PCR primers for GETV genome fragments.
