# Supplementary figures and images for: Exosome-enriched extracellular vesicles are associated with Getah virus RNA and transmission-related readouts in vitro
Source: Front Cell Infect Microbiol. 2026 Jun 15;16:1838901. doi: 10.3389/fcimb.2026.1838901 (PMC13311032; doi:10.3389/fcimb.2026.1838901)

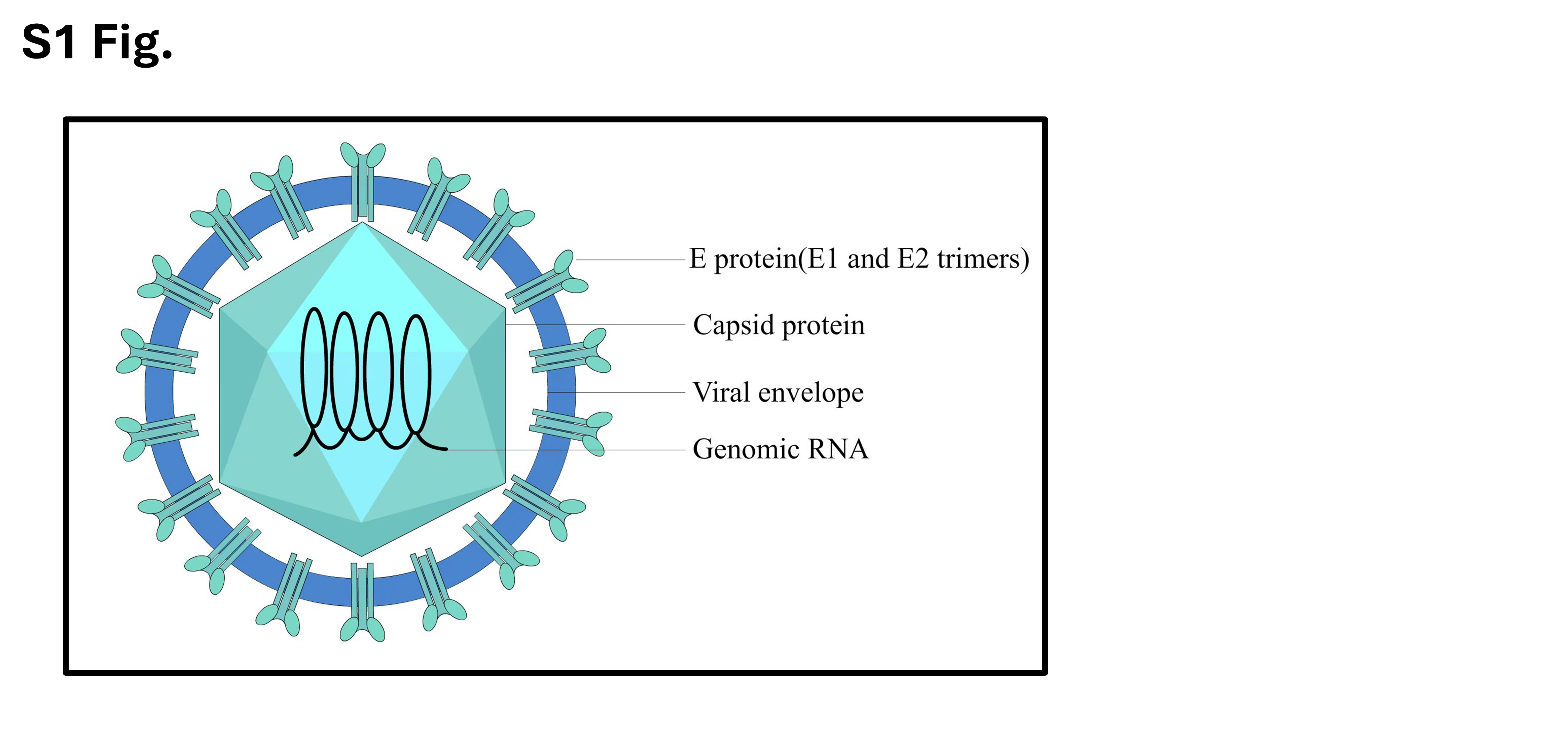

Supplement: Supplementary Figure 1 — GETV virion structure. [file Image1.tif]

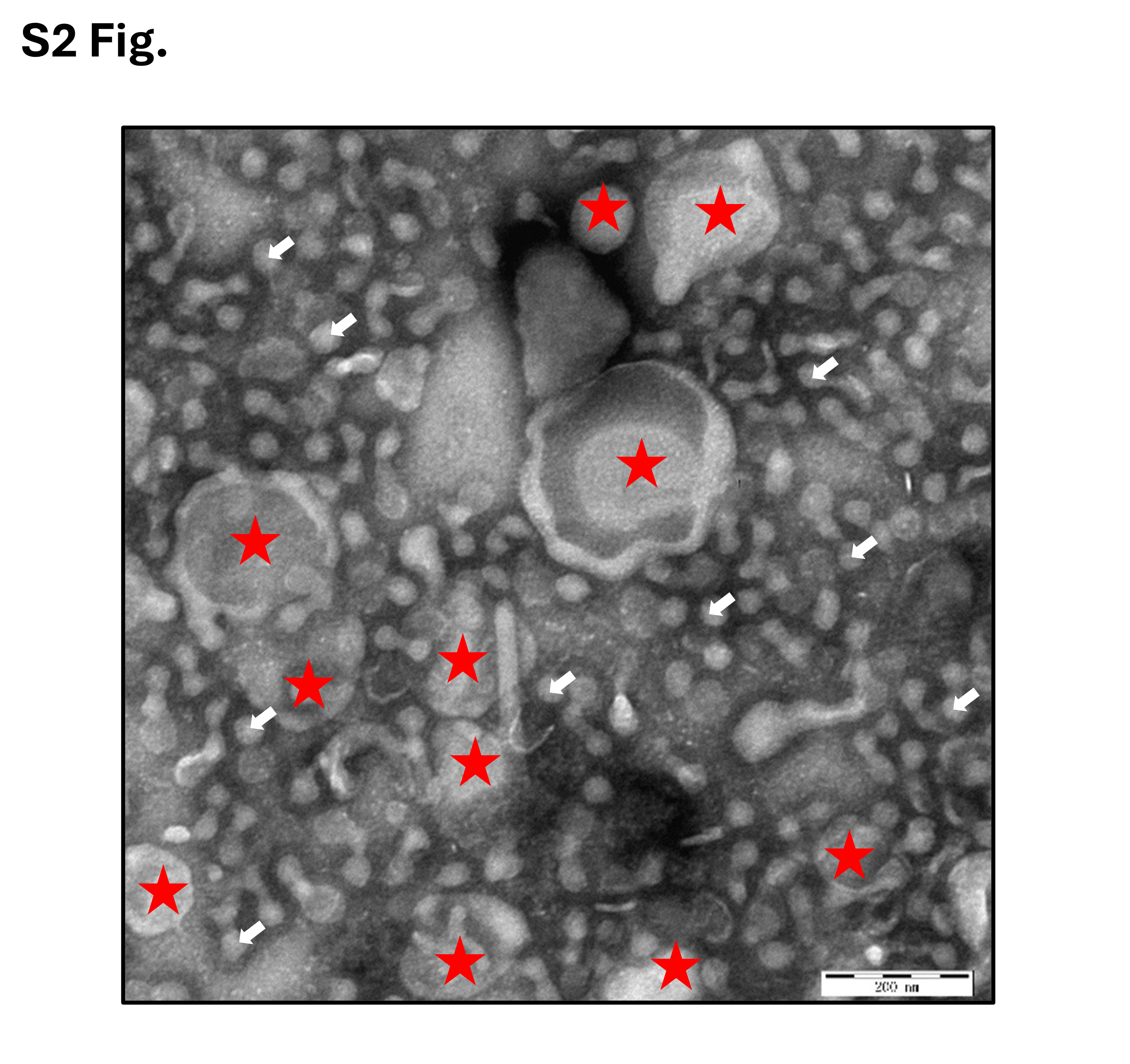

Supplement: Supplementary Figure 2 — TEM of negatively stained crude EV pellets. [file Image2.tif]

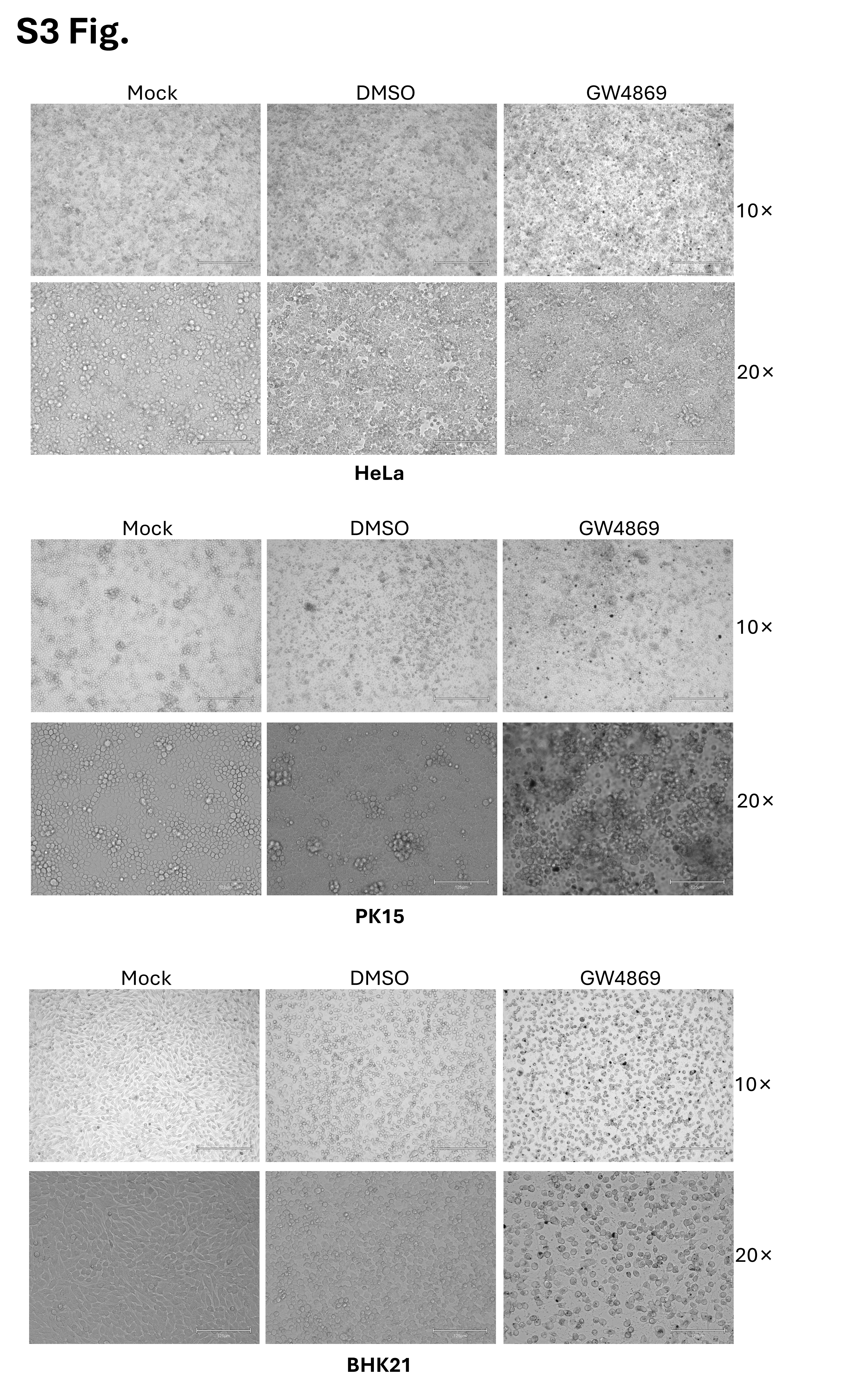

Supplement: Supplementary Figure 3 — GW4869 effects in different cell lines. [file Image3.tif]

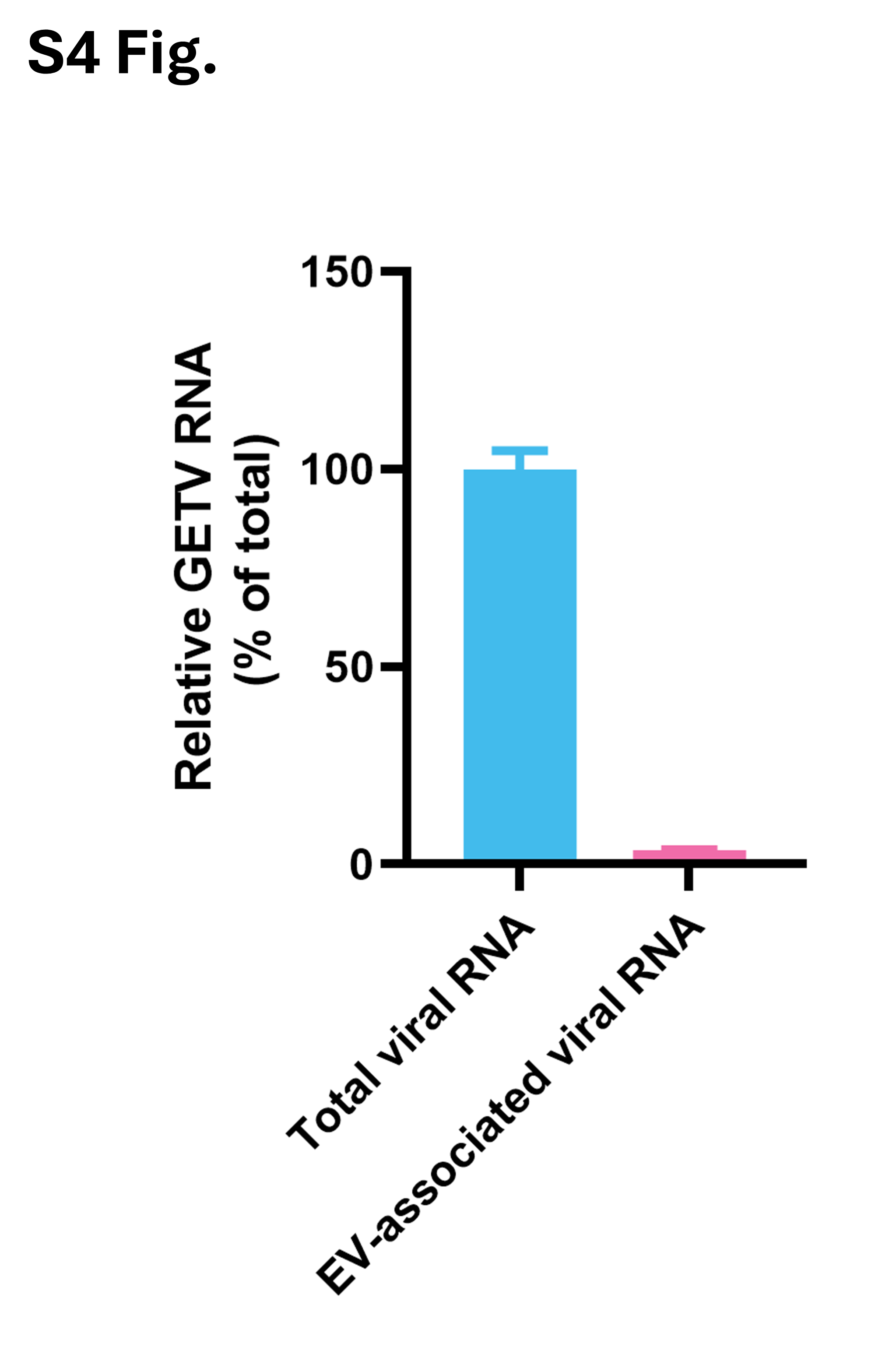

Supplement: Supplementary Figure 4 — Quantification of the proportion of total extracellular GETV RNA with exosome-enriched EV preparations. [file Image4.tif]

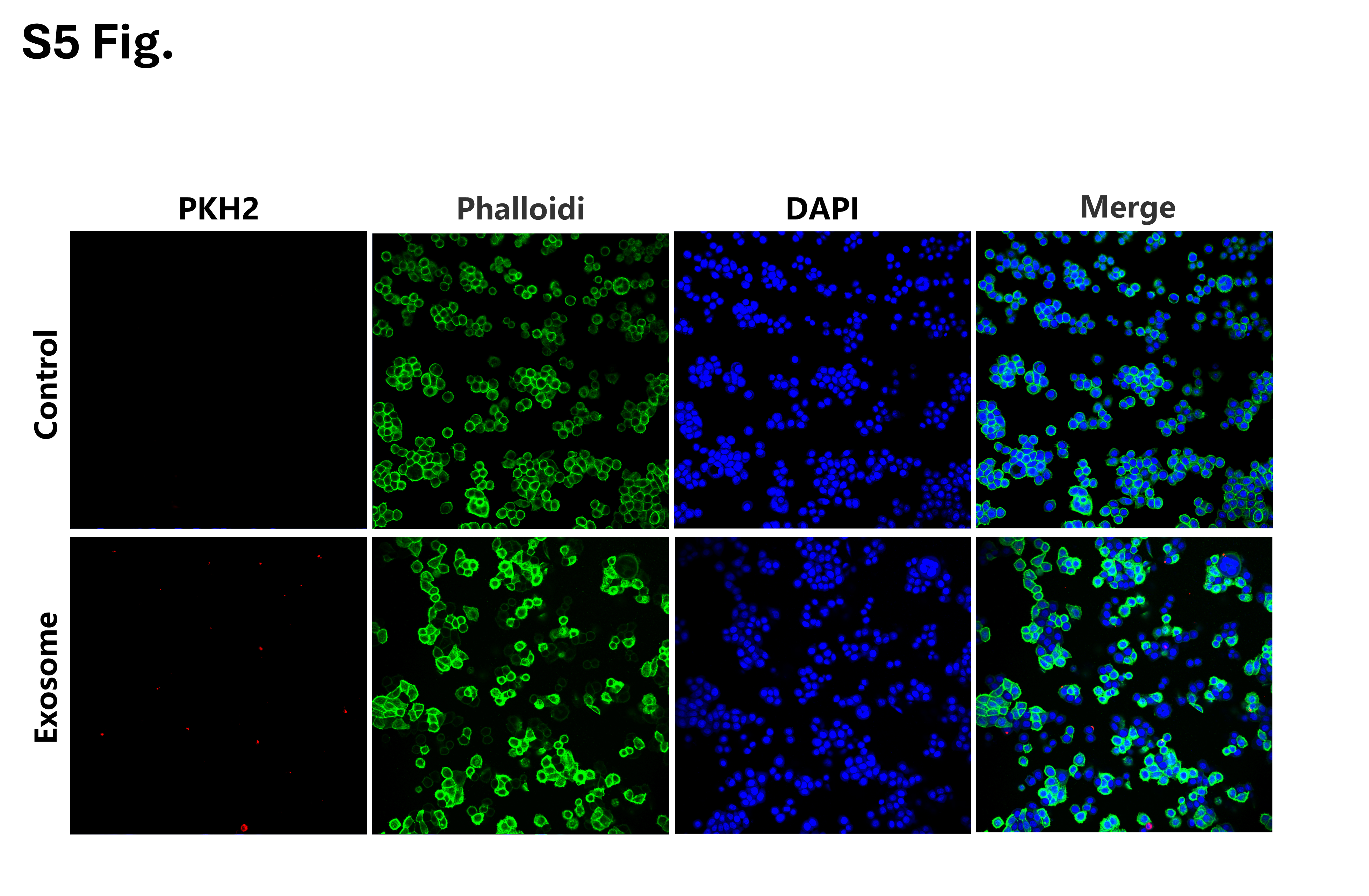

Supplement: Supplementary Figure 5 — Wider-field representative images of PKH26-labeled exosome-enriched EV uptake by recipient HeLa cells. [file Image5.tif]

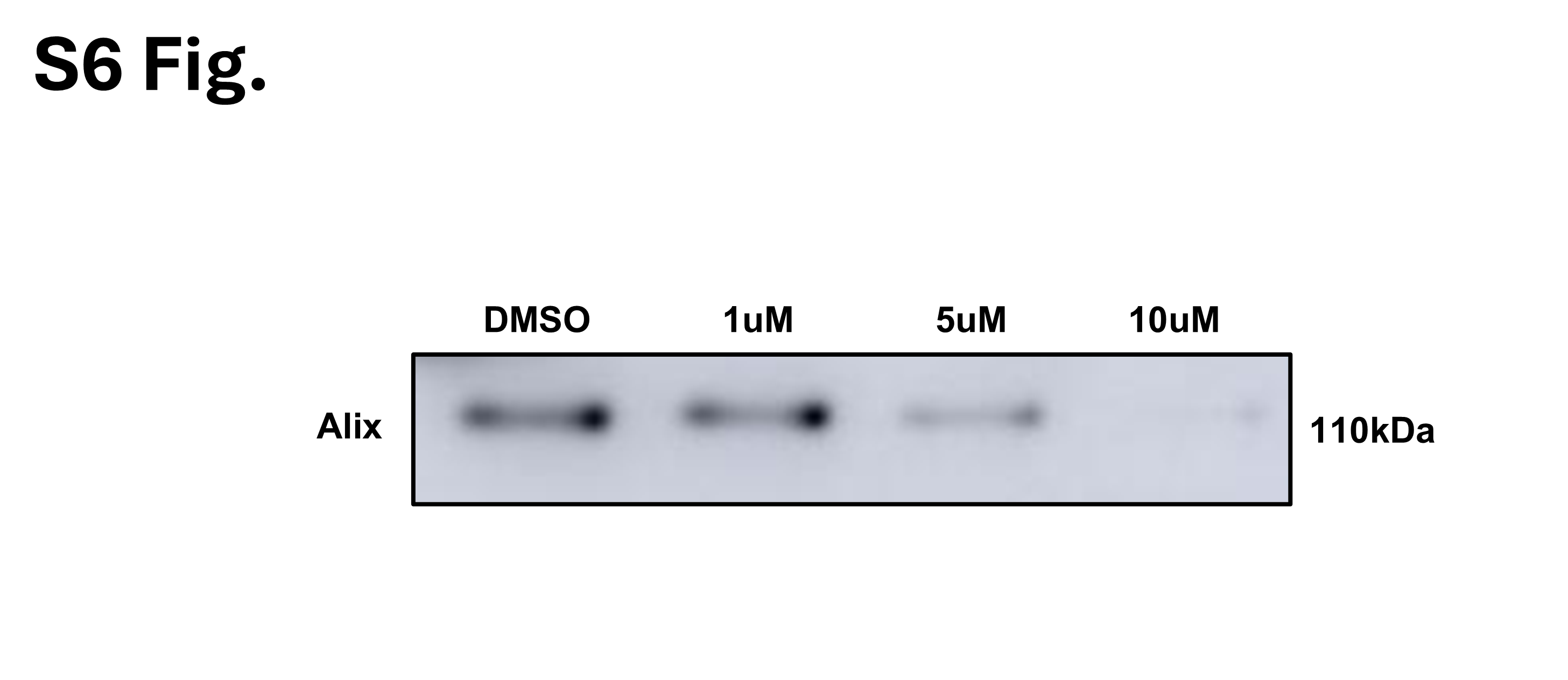

Supplement: Supplementary Figure 6 — GW4869 treatment is associated with reduced recovery of Alix in exosome-enriched EV preparations. [file Image6.tif]
