# Supplementary material for: Exosome-enriched extracellular vesicles are associated with Getah virus RNA and transmission-related readouts in vitro
Source: Front Cell Infect Microbiol. 2026 Jun 15;16:1838901. doi: 10.3389/fcimb.2026.1838901 (PMC13311032; doi:10.3389/fcimb.2026.1838901)
Supplement: Supplementary Table 1 — RT-PCR primers for GETV genome fragments. [file Table1.docx]

Table S1. RT-PCR primers for GETV genome fragments.

| Primer | Sequence (5’-3’) |
| --- | --- |
| D1.GETV-1U21 | ATG GCG GAC GTG TGA CAT CAC |
| D2.GETV-659L23 | CTC ATC TGC CCA GTT GGT CGA GT |
| D3.GETV-518U23 | CCG TGT ACC AGG ACG TAT ACG CT |
| D4.GETV-1940L23 | ATT GTA CAC CAT GGT TGC GCT CT |
| D5.GETV-1804U23 | CAC CCT CTT GCT GAG CAA GTC AC |
| D6.GETV-3314L23 | AGG CCT GTT GTC CCA GTG GTT GT |
| D7.GETV-3142U23 | GAG ACG GCC GGA ATA CGT ATG AC |
| D8.GETV-4619L23 | ACC ATT TCT GCC GAC TAA GCA AC |
| D9.GETV-4485U23 | AGA CGT GGT TAT CTA CTG CAG AG |
| D10.GETV-5943L23 | ACA CAG GTC TAG GGT ACT TGC TG |
| D11.GETV-5807U23 | TGG CCC CGA CAG AAG CGA ATA AG |
| D12.GETV-7262L23 | TCT GTC TTC ATC CTG GTT GTC GT |
| D13.GETV-7121U23 | AGA TCA TCG ATG CCA CAA TGT GT |
| D14.GETV-8600L23 | TAG CCA CCG GGC TGT AAC AGA AC |
| D15.GETV-8413U23 | AAC AAC CAG AAC AGA CAC TGA GG |
| D16.GETV-9872L23 | GGC AGC ATA CAA GTA TGA TTA TG |
| D17.GETV-9660U23 | TCG CTG CTG GCG TCA TGT TAC AT |
| D18.GETV-11123L21 | CCA TAA GGG ACG ATG TGA TCC |
| D19.GETV-11015U23 | AGA TGG CAA GAT AAC CCT GCA TT |
| D20.GETV-polyAL34 | GCC CGC GCA TGC GAT ATC TTT TTT TTT TTT TTT T |
